# Advancing understanding and identifying strategies for sustaining evidence-based practices: a review of reviews

**DOI:** 10.1186/s13012-020-01040-9

**Published:** 2020-10-09

**Authors:** Sarah A. Birken, Emily R. Haines, Soohyun Hwang, David A. Chambers, Alicia C. Bunger, Per Nilsen

**Affiliations:** 1 Department of Health Policy and Management, Gillings School of Global Public Health, The University of North Carolina at Chapel Hill, 1103E McGavran-Greenberg, 135 Dauer Drive, Campus Box 7411, Chapel Hill, NC 27599-7411 USA; 2 Department of Health Policy and Management, The University of North Carolina at Chapel Hill, 1101B McGavran - Greenberg Hall, CB# 7411, Chapel Hill, NC 27599-7411 USA; 3 Division of Cancer Control and Population Sciences, National Cancer Institute, 9609 Medical Center Drive, Room 3E414, Rockville, MD 20850 USA; 4 College of Social Work, The Ohio State University, 1947 College Road, Columbus, OH 43210 USA; 5 Department of Medical and Health Sciences, Division of Community Medicine, Linköping University, SE-581 83 Linköping, Sweden

**Keywords:** Sustainment, Sustainability, Systematic reviews, Theories, models, and frameworks

## Abstract

**Background:**

Implementation science has focused mainly on the initial uptake and use of evidence-based practices (EBPs), with less attention to sustainment—i.e., continuous use of these practices, as intended, over time in ongoing operations, often involving adaptation to dynamic contexts. Declining EBP use following implementation is well-documented yet poorly understood. Using theories, models, and frameworks (TMFs) to conceptualize sustainment could advance understanding. We consolidated knowledge from published reviews of sustainment studies to identify TMFs with the potential to conceptualize sustainment, evaluate past uses of TMFs in sustainment studies, and assess the TMFs’ potential contribution to developing sustainment strategies.

**Methods:**

We drew upon reviews of sustainment studies published within the past 10 years, evaluated the frequency with which included articles used a TMF for conceptualizing sustainment, and evaluated the relevance of TMFs to sustainment research using the Theory, Model, and Framework Comparison and Selection Tool (T-CaST). Specifically, we examined whether the TMFs were familiar to researchers, hypothesized relationships among constructs, provided a face-valid explanation of relationships, and included sustainment as an outcome.

**Findings:**

Nine sustainment reviews referenced 648 studies; these studies cited 76 unique TMFs. Only 28 TMFs were used in more than one study. Of the 19 TMFs that met the criteria for T-CaST analysis, six TMFs explicitly included sustainment as the outcome of interest, 12 offered face-valid explanations of proposed conceptual relationships, and six identified mechanisms underlying relationships between included constructs and sustainment. Only 11 TMFs performed adequately with respect to all these criteria.

**Conclusions:**

We identified 76 TMFs that have been used in sustainment studies. Of these, most were only used once, contributing to a fractured understanding of sustainment. Improved reporting and use of TMFs may improve understanding of this critical topic. Of the more consistently used TMFs, few proposed face-valid relationships between included constructs and sustainment, limiting their ability to advance our understanding and identify potential sustainment strategies. Future research is needed to explore the TMFs that we identified as potentially relevant, as well as TMFs not identified in our study that nonetheless have the potential to advance our understanding of sustainment and identification of strategies for sustaining EBP use.

Contributions to the literature

- Our review identified theories, models, and frameworks used in sustainment research.
- Our review assessed the relevance of theories, models, and frameworks that have been used in extant sustainment research for advancing the understanding of sustainment.
- Our review identified theories that explain relationships among included constructs and hence may help inform strategies for sustainment in practice.

## Background

Implementation science has emerged as a vital, multidisciplinary research field in the wake of the evidence-based movement [1]. Thus far, research in the field has focused mainly on identifying factors affecting the initial uptake and use of evidence-based practices (EBPs). Less attention has been given to the sustainment of implemented practices—i.e., continuous evidence-based practice (EBP) use, as intended, over time in ongoing operations, often involving adaptation to dynamic contexts [2–4]. Many EBPs are adopted, only for their benefit to wane [5–7]. The declining quality, intensity, and comprehensiveness of EBP use following implementation are well-documented [8–10], but how and why EBP use is sustained remains unclear.

There are several reasons for the limited knowledge about EBP sustainment. First, there is a lack of conceptual clarity in the literature [11]: Researchers use a range of terms to describe sustainment, including sustainability, which is a related, but distinct, term that refers to preparedness for sustained use or the characteristics of a new practice which will enhance its sustainment [12, 13]. Other terms that may reflect an ongoing process expected to result in sustainment include, for example, continuation, durability, institutionalization, sustained use, and routinization [8]. Hence, it is possible that the same underlying concept (i.e., sustainment) is described using different terms (synonymy) or the same key terms might be defined in different ways (polysemy). The use of imprecise concepts and terms makes knowledge exchange and learning on this topic difficult. Second, there are methodological challenges to studying sustainment: The post-implementation duration required to achieve sustainment is unclear [8], and the period required for assessing sustainment may exceed grant funding periods. Third, knowledge regarding EBP sustainment may be limited by the lack of an agreed-upon theory, model, or framework (hereafter referred to in combination as TMFs).

Using TMFs to conceptualize sustainment could alleviate issues related to synonymy and polysemy and offer a structure for organizing and comparing findings across study settings. In addition, TMFs could help guide all phases of sustainment research and practice, including assessment of the form and degree of sustainment, identification of determinants of sustainment, selection of strategies for promoting sustainment, and evaluation of sustainment-related outcomes [14]. In particular, theories (in contrast to models and frameworks, which do not specify theoretical relationships in a way that explains how or why EBPs are sustained) are needed to suggest strategies for promoting EBP sustainment [2, 13].

The extent to which TMFs are used to advance understanding of sustainment is unclear. We consolidated knowledge from published reviews of healthcare sustainment studies to identify TMFs with the potential to conceptualize sustainment, evaluate past uses of these TMFs in sustainment studies, and assess the TMFs’ potential contribution to the development of sustainment strategies. A recent systematic review by Penno and colleagues identified and analyzed existing TMFs that focus on the sustainability of EBPs in specific healthcare settings [15]. Penno and colleagues’ review examined the concepts and factors associated with sustainability within the TMFs (i.e., *what* influences sustainment). Our review complements Penno and colleagues’ review by identifying TMFs that explain the relationships included among constructs to inform sustainment strategies (i.e., *why* constructs are thought to influence sustainment). We conclude by recommending TMFs that have the greatest potential to advance understanding of sustainment in future research and thereby may contribute to identifying potential strategies for EBP sustainment.

## Method

### Search strategy

To identify TMFs that have been used in healthcare sustainment research, we drew upon recently published reviews of sustainment studies. To identify reviews, we followed an approach similar to that of Moore et al.’s search for knowledge syntheses of sustainability in healthcare interventions [16]: We used the PubMed search filter for reviews to identify articles with the terms “sustainability,” “sustainment,” “durability,” “institutionalization,” “routinization,” “continuation,” or “sustained” in the title and published in the past 10 years. We used this range of terms to account for the common use of different terms to describe the concept of sustainment [8].

### Inclusion criteria

To be included, we required articles to (1) be written in English, (2) be published in the past 10 years, (3) review articles of sustainment studies, and (4) report on the use of TMFs among included studies.

### Study selection

Three authors selected records for inclusion in the study. These authors conducted title, abstract, and full-text review, searching for inclusion of sustainment as a key construct of interest. The three authors resolved discrepancies through discussions, and they reached consensus. Two authors then reviewed the full text of the remaining articles, confirming evidence of reviews of sustainment studies with reports of TMFs in each record.

### Data abstraction and analysis

#### TMFs used in sustainment studies

We reported the frequency with which included articles used a TMF for conceptualizing sustainment, identified all TMFs used by the studies included in the selected review articles, and reported the prevalence of the TMFs across studies. Although they reported on the use of TMFs in studies they included, Shigayeva and Coker (2015) [17] did not indicate the number of included studies that used TMFs, so we reviewed all 108 empirical studies included in Shigayeva and Coker (2015) [17]. From those studies, we abstracted information regarding whether a TMF was used and, if so, which TMF. Two authors independently reviewed 20% (*n* = 22/108 empirical studies included in Shigayeva and Coker (2015) [17]) of the articles to ensure reliability in abstraction; a single investigator abstracted information from the remaining articles. We excluded articles reporting framework development.

#### Relevance of TMFs for understanding sustainment

To evaluate the relevance of TMFs for understanding sustainment, we evaluated the TMFs using four criteria: degree of familiarity of the TMF to researchers, operationalized in terms of frequency or usage, and three additional criteria from the Theory, Model, and Framework Comparison and Selection Tool (T-CaST) [18], a user-friendly tool designed to help select TMFs for implementation research. T-CaST guides researchers through rating candidate TMFs’ performance with respect to four domains: usability, testability, applicability, and acceptability. Our study objectives were to identify TMFs that could advance understanding of sustainment and contribute to identifying strategies for sustainment. To achieve these objectives, two authors used T-CaST to rate the performance of TMFs identified in the review from their own perspectives with respect to the following criteria:

1. TMF is familiar to key stakeholders (i.e., implementation researchers). We defined familiarity as being used in two or more articles.
2. TMF provides an explanation of how included constructs influence sustainment and/or each other (i.e., TMF hypothesizes relationships among constructs rather than simply listing them).
3. TMF includes meaningful, face-valid explanations of proposed relationships (i.e., hypothesized relationships among constructs are logically consistent and plausible).
4. TMF includes sustainment as an outcome.

Application of the first criterion left 28 TMFs we then evaluated in terms of criteria (2)–(4).

T-CaST rates TMFs 0, 1, or 2 with respect to relevant criteria, where 0 = TMF does not fit criterion, 1 = TMF fits criterion moderately well, and 2 = TMF fits criterion well. Thus, after limiting TMFs to the 28 cited by two or more included articles, a TMF could score from 0 (TMF does not fit any selected criteria well) to 6 (TMF fits criteria well). We excluded nine of the 28 remaining TMFs because they were too broad in scope (e.g., “theories of organizational change and innovation”), too vague to identify (e.g., “intervention theory”), or were not TMFs (e.g., “child survival sustainability assessment”). Two authors, trained implementation scientists, independently coded nine TMFs and resolved discrepancies through discussions until they reached consensus; they then individually coded the remaining 10 TMFs.

## Results

### Search results

**Fig. 1:**
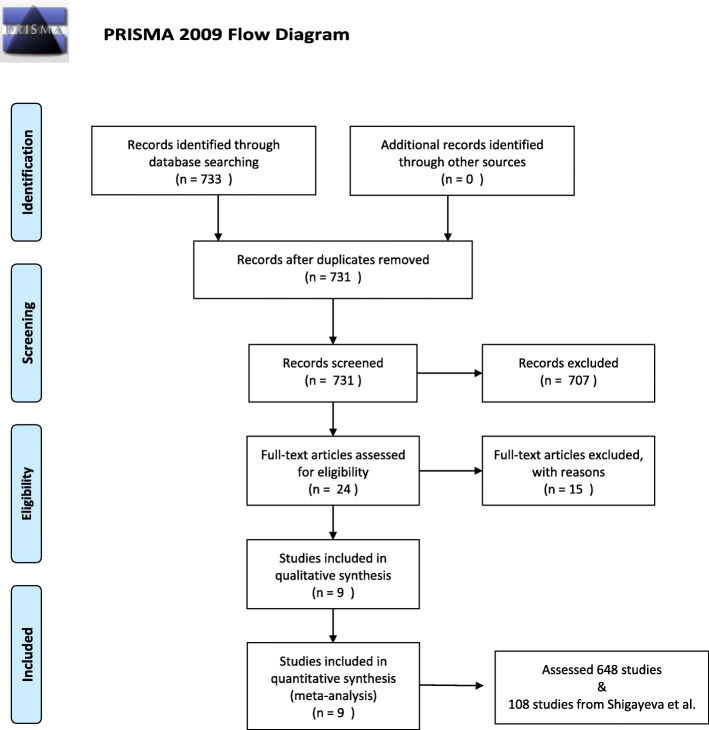
PRISMA 2009 flow diagram

This search yielded 733 publications. Of these, 709 publications were excluded because they were not published in English, were not published in the past 10 years, did not review sustainment studies, and/or did not report on TMF use among included studies. A subsequent review of article titles and abstracts narrowed this down to 24 publications deemed relevant. Upon full-text review, 15 additional articles were excluded because, upon closer inspection, they did not meet inclusion criteria. There remained nine review articles which contained information about 648 empirical studies of sustainment in healthcare settings. This process is summarized in Fig. 1 [8, 17, 19–25].

### Description of included reviews

Table 1 displays characteristics of included reviews (systematic, scoping, and literature reviews) spanning multiple fields and disciplines, such as global health [27, 28], chronic disease [19], and communicable disease [29]. Collectively, the reviews covered from 1979 to 2017. Four reviews had indicated the quality appraisal of the articles. Six of the nine reviews were explicitly limited to English language articles published. Four of the nine reviews reported formally assessing the quality of the included articles.

**Table 1:** Reviews of sustainment studies

Authors (year)	Years covered	Language	Quality appraisal	Review type	Interventions of interest	Proportion of review’s included studies reporting TMF use
Tricco et al. (2016) [19]	1979–2012	Unspecified	No	Scoping	Chronic disease management	“[N]one of the included studies reported using a framework to develop, implement, or measure sustainability” (p.5). [0/144]
Wiltsey Stirman et al. (2012) [8]	Published or in press by July 2011	English	No	Systematic	Various medical care and health services, mental and behavioral health, health promotion and public health, and education	“[F]ewer than one-third of the studies that we reviewed were guided by an explicit model” (p.12) [1/3 × 125 = 41/125]
Hulland et al. 2015 [20]	Published or available by December 1, 2013	English, French, German, or Spanish	Yes	Systematic	Water, hygiene, and sanitation	“[O]nly 11 of the 36 studies described a behavioral model or conceptual framework” (p.44). [11/36 articles]
Lovarini et al. (2013) [21]	Unspecified	English	Yes	Systematic	Community-based fall prevention	“Three publications described different conceptual frameworks or models of program sustainability” (p.11). [3/19]
Hodge and Turner (2016) [24]	Unspecified	English	No	Literature	Various for disadvantaged communities	“Only 11 of the articles indicated that they were guided by a conceptual framework for implementation” (p.196). [11/28]
Iwelunmor et al. (2016) [22]	1996–2015	English	Yes	Systematic	Various implemented in Sub-Saharan Africa	“[Twenty-three] of the 41 articles reviewed discussed framing the sustainability in terms of a theory or conceptual framework” (p.15). [23/41]
Schell et al. (2013) [23]	Literature spans about 20 years	Unspecified	No	Literature	Various in public health	“Some pieces highlighted the relevance of institutional theory, Schien’s work on organizational culture, or diffusion of innovations” (p.5). [3/85]
Lennox et al. (2018) [26]	Final search conducted September 2017	English	Yes	Systematic	Various in health care (i.e., models, checklists, tools, processes, strategies, conceptualizations and frameworks)	“37% (23/62) did not have an explicit link to theory” (p.4). [39/62]
Shigayeva and Coker (2015) [17]	1980–2012	English	No	Literature	Communicable disease programs	66% [71/108] of empirical studies included in the review did not use a TMF. [37/108]

*TMF* theory, model, and/or framework

### TMF use

Seven of the nine identified reviews reported the proportion of included studies that explicitly used a TMF [8, 17, 19–24]; an estimated 194/478 (41%) of the studies included in these seven reviews used a TMF to study sustainment (see Table 1 for details on estimate).

The remaining two reviews focused exclusively on articles that used TMFs to study sustainment [26, 29]. For example, Lennox et al. [26] reviewed the literature for publications reporting the use of TMFs (i.e., models, checklists, tools, processes, strategies, conceptualizations, and frameworks) and found that 37% (23/62) of the included studies did not report using a theory, although the included studies may have used models and/or frameworks.

Table 2 reports the 76 unique TMFs cited in the 648 studies included across the 9 reviews. The most frequently cited TMFs were diffusion of innovations (*n* = 16), ecological theories (*n* = 10), complexity theory (*n* = 10), and normalization process theory (*n* = 6). Most of the TMFs that were cited across the 9 reviews (48/76) were only used by one of the 648 studies.

**Table 2:** TMF cited in included studies

	TMF	Number of studies citing TMF*	Review article (number of included studies citing TMF)
1	Diffusion of innovations theory	16	Schell et al. 2013 (1) [23]; Lovarini et al. 2013 (1) [21], Lennox et al. 2018 (10) [26]; Shigayeva and Coker 2015 (2) [17]; Iwelunmor et al. 2016 (1) [22]; Hulland et al. 2015 (1) [20]
2	Ecological theories	10	Lennox et al. 2018 (5) [26]; Shigayeva and Coker 2015 (2) [17]; Iwelunmor et al. 2016 (3) [22]
3	Complexity theory	10	Lennox et al. 2018 (9) [26]; Hodge and Turner 2016 (1) [24]
4	Normalization process theory	6	Lennox et al. 2018 (3) [26]; Shigayeva and Coker 2015 (3) [17]
5	Model of institutionalization	6	Shigayeva and Coker 2015 (5) [17]; Hodge and Turner (1) [24]
6	Open systems theories	5	Lennox et al. 2018 (4) [26]; Shigayeva and Coker 2015 (1) [17]
7	Conceptual framework on sustainability	5	Iwelunmor et al. 2016 (3) [22]; Shigayeva and Coker 2015 (2) [17]
8	Dynamic sustainability framework	4	Iwelunmor et al. 2016 (4) [22]
9	Theories of organizational change and innovation	3	Shigayeva and Coker 2015 (3) [17]
10	Organizational theory: formation of inter-organizational relationships	3	Shigayeva and Coker 2015 (3) [17]
11	Institutional theory	3	Schell et al. 2013 (1) [23]; Lennox et al. 2018 (1) [26]; Shigayeva and Coker 2015 (1) [17]
12	Continuous quality improvement	3	Lennox et al. 2018 (3) [26]
13	Organizational learning theory	3	Lennox et al. 2018 (1) [26]; Shigayeva and Coker 2015 (2) [17]
14	World Health Organization guidelines and models	3	Hodge and Turner (1) [24]; Iwelunmor et al. 2016 (2) [22]
15	Theory of planned behavior/theory of reasoned action	3	Lennox et al. 2018 (1) [26]; Martin et al. 2018 (1) [30]; Shigayeva and Coker 2015 (2) [17]
16	Social learning theory/social cognitive theory	3	Shigayeva and Coker 2015 (3) [17]
17	Network theory	3	Lennox et al. 2018 (2) [26]; Shigayeva and Coker 2015 (1) [17]
18	Health belief model	2	Hulland et al. 2015 (2) [20]
19	Child Survival Sustainability Assessment framework	2	Lennox et al. 2018 (1) [26]; Shigayeva and Coker 2015 (1) [17]
20	Freire’s conscientization theory	2	Iwelunmor et al. 2016 (1) [22]; Shigayeva and Coker 2015 (1) [17]
21	Program Sustainability Index	2	Hodge and Turner (2) [24]
22	Framework for the assessment of sustainability	2	Iwelunmor et al. 2016 (1) [22]; Shigayeva and Coker 2015 (1) [17]
23	System dynamics	2	Lennox et al. 2018 (1) [26]; Shigayeva and Coker 2015 (1) [17]
24	Theory of organization routines	2	Lennox et al. 2018 (1) [26]; Shigayeva and Coker 2015 (1 )[17]
25	HIV/AIDS Program Sustainability Analysis Tool	2	Iwelunmor et al. (1) [22]; Shigayeva and Coker 2015 (1) [17]
26	Sustainability planning model	2	Iwelunmor et al. (1) [22]; Shigayeva and Coker 2015 (1) [17]
27	Sustainability framework for community-based dengue control projects	2	Hodge and Turner (1) [24]; Shigayeva and Coker 2015 (1) [17]
28	Organizational sustainability framework	2	Shigayeva and Coker 2015 (2) [17]
29	Organizational culture	1	Schell et al. 2013 [23]
30	Intervention (program) theory	1	Lennox et al. 2018 [26]
31	Focus on opportunity, ability, and motivation	1	Hulland et al. 2015 [20]
32	Risk, attitude, norm, ability, self-regulation	1	Hulland et al. 2015 [20]
33	PATH’s Behavior Change Continuum	1	Hulland et al. 2015 [20]
34	Transtheoretical model of change	1	Hulland et al. 2015 [20]
35	Consumer purchase decision process	1	Hulland et al. 2015 [20]
36	Elaboration of likelihood	1	Hulland et al. 2015 [20]
37	Dimensions of social research	1	Hulland et al. 2015 [20]
38	Knowledge dissemination and utilization framework	1	Lovarini et al. 2013 [21]
39	Policy, research, and service delivery model for fall prevention	1	Lovarini et al. 2013 [21]
40	Organizational theory	1	Lovarini et al. 2013 [21]
41	Systems thinking-guided analysis framework	1	Iwelunmor et al. 2016 [22]
42	Model of motivational processes	1	Iwelunmor et al. 2016 [22]
43	Clinical assessment for systems strengthening framework	1	Iwelunmor et al. 2016 [22]
44	“Train the trainer” model	1	Iwelunmor et al. 2016 [22]
45	Community-based management of acute malnutrition of the Belgian Red Cross	1	Iwelunmor et al. 2016 [22]
46	Organizational readiness to change theory	1	Iwelunmor et al. 2016 [22]
47	In-service training improvement framework	1	Iwelunmor et al. 2016 [22]
48	Promoting school-community-university partnerships to enhance resilience model	1	Hodge and Turner 2016 [24]
49	Evaluation theory	1	Lennox et al. 2018 [26]
50	Model for improvement	1	Lennox et al. 2018 [26]
51	Adaptive management	1	Lennox et al. 2018 [26]
52	Evidence integration triangle	1	Lennox et al. 2018 [26]
53	Self-determination theory	1	Lennox et al. 2018 [26]
54	Theory of change	1	Lennox et al. 2018 [26]
55	Absorptive capacity	1	Lennox et al. 2018 [26]
56	Dartmouth psychiatric research center implementation model	1	Hodge and Turner 2016 [24]
57	School-wide positive behavior support continuum	1	Hodge and Turner 2016 [24]
58	Exploration, planning, implementation, sustainment	1	Hodge and Turner 2016 [24]
59	Community readiness model	1	Shigayeva and Coker 2015 [17]
60	Theory of how to design effective organizations	1	Shigayeva and Coker 2015 [17]
61	Reach effectiveness adoption implementation maintenance	1	Shigayeva and Coker 2015 [17]
62	Model of community-based program sustainability	1	Shigayeva and Coker 201 5[31]
63	Precede framework	1	Shigayeva and Coker 2015 [17]
64	Communities that Care framework	1	Shigayeva and Coker 2015 [17]
65	World Health Organization safe community model	1	Shigayeva and Coker 2015 [17]
66	National Funding Collaborative on Violence Prevention’s Theory of Change	1	Shigayeva and Coker 2015 [17]
67	STEP-UP framework	1	Shigayeva and Coker 2015 [17]
68	Conceptual model of social determinants of health	1	Shigayeva and Coker 2015 [17]
69	Sustainability benchmarks	1	Shigayeva and Coker 2015 [17]
70	Nature of partnerships	1	Shigayeva and Coker 2015 [17]
71	Five basic elements of program sustainability for tobacco control programs	1	Shigayeva and Coker 2015 [17]
72	Mandiana model	1	Shigayeva and Coker 2015 [17]
73	Sustainability checklist	1	Shigayeva and Coker 2015 [17]
74	Scheirer’s framework to assess the development and capacity of non-profit agencies	1	Shigayeva and Coker 2015 [17]
75	Punctuated equilibrium theory	1	Shigayeva and Coker 2015 [17]
76	Multi-level model of factors to be identified at the levels of the innovation	1	Shigayeva and Coker 2015 [17]

*TMF* theory, model, and/or framework

*Across 648 studies included in the nine reviews

### Relevance of TMFs used in sustainment studies

The TMFs that we scored received ratings ranging from 2 to 5 (see Table 3). The TMF with the highest score was institutional theory, which scored 5 out of 6. TMFs scoring 4 out of 6 included the model of institutionalization [31], diffusion of innovations theory [32], open systems theories [33], normalization process theory [29], organizational learning theory [34], the health belief model [27], network theory [35], the theory of planned behavior [28], the organizational sustainability framework [36], and the theory of organization routines [37].

**Table 3:** Relevance of sustainment TMF

	TMF	Provides an explanation of how included constructs influence sustainment and/or each other	Includes meaningful, face-valid explanations of proposed relationships	Includes sustainment as an outcome	Overall T-CaST score	Notes
1	Institutional theory	2	2	1	5/6	Institutional theory enhances understanding the organizations’ practice sustainment in response to three key pressures but offers limited insight into potentially influential factors at inner setting and individual levels. Its outcome is isomorphism (i.e., increasing likeness), which may be related to sustainment but is conceptually distinct.
2	Model of institutionalization	1	2	1	4/6	The model of institutionalization identifies six factors associated with institutionalization (e.g., standard operating routines; program champion actions). It offers face-valid explanations of proposed relationships, but it lacks a description of the mechanisms underlying those relationships, and its outcome is institutionalization (i.e., “the final stage of an innovation-diffusion process”), which may be related to sustainment but is conceptually distinct.
3	Diffusion of innovations theory	2	2	0	4/6	Diffusion of innovations theory explains how people, as part of a social system, adopt a new idea, behavior, or product through five established adopter categories: innovators, early adopters, early majority, late majority, and laggards. It offers face-valid explanations of proposed relationships but lacks discrete constructs that might be operationalized as antecedents to sustainment, and its outcome is innovation diffusion, which is conceptually distinct from sustainment.
4	Open systems theories	0	2	2	4/6	Open systems theories broadly propose that organizations are strongly influenced by their environments. They offered a meaningful, face-valid explanation of sustainment but do not include discrete constructs, thereby limiting our ability to operationalize or falsify the theory. Further, open systems theories are an umbrella that encompasses several theories, not a singular TMF.
5	Normalization process theory	2	1	1	4/6	Normalization process theory describes the social processes leading the routinization of EBPs. It explains relationships among included constructs but does not offer a clear conceptual distinction between “integration”/”embeddedness” and implementation.
6	Organizational learning theory	1	2	1	4/6	Organizational learning theory describes a process of organizations embedding knowledge from experience. It offers face-valid explanations of proposed conceptual relationships but lacks discrete constructs that might be operationalized as antecedents to sustainment, and its outcome is knowledge, which may be related but is conceptually distinct from sustainment.
7	Health belief model	2	2	0	4/6	The health belief model theorizes that people’s beliefs about whether or not they are at risk for a disease or health problem and their perceptions of the benefits of taking action to reduce or avoid influence their readiness to take action. It offers face-valid explanations of proposed conceptual relationships and identifies mechanisms underlying relationships between included constructs and the outcome; however, its outcome is action, which is conceptually distinct from sustainment.
8	Network theory	2	2	0	4/6	Network theory advances understanding how extant networks affect either the flow of information and resources to individual actors or how individual actors gain prestige or influence through their positions in networks. It offers face-valid explanations of proposed conceptual relationships and identifies mechanisms underlying relationships between included constructs and the outcome; however, its outcome is relational connections, which is conceptually distinct from sustainment.
9	Theory of planned behavior	2	2	0	4/6	The theory of planned behavior offers face-valid explanations of proposed conceptual relationships and identifies mechanisms underlying relationships between included constructs and the outcome; however, its outcome is behavior, which may be related but is conceptually distinct from sustainment.
10	Organizational sustainability framework	1	1	2	4/6	The organizational sustainability framework suggests that sustainability, a term that is related yet distinct from sustainment, is a function of economic, environmental, and social organizational sustainability. The framework identifies very general mechanisms underlying relationships between included constructs and the outcome, and the constructs that it includes are somewhat meaningful and face-valid if not comprehensive.
11	Theory of organization routines	1	2	1	4/6	The theory of organization routines suggests that routines are developed through directions and performances among organizational members. It identifies meaningful, face-valid constructs hypothesized to facilitate routines, but it does not specify the mechanisms underlying the relationships, and its outcome is routines (i.e., ways of accomplishing organizational work), which may be related to sustainment but is conceptually distinct.
12	Complexity theory	0	2	1	3/6	
13	Dynamic sustainability framework	0	1	2	3/6	
14	Freire’s conscientization theory	1	1	1	3/6	
15	Sustainability planning model	0	1	2	3/6	
16	Social learning theory/social cognitive theory	1	2	0	3/6	
17	Ecological theories	0	2	0	2/6	
18	Program Sustainability Index	0	0	2	2/6	
19	Sustainability framework for community-based dengue control projects	0	0	2	2/6	
20	Theories of organizational change and innovation				[Eliminated (too broad)]	
21	Organizational theory: formation of inter-organizational relationships				[Eliminated (too broad)]	
22	Conceptual framework on sustainability				[Eliminated (insufficiently specified)]	
23	Continuous quality improvement				[Eliminated (too broad)]	
24	World Health Organization guidelines and models				[Eliminated (too broad)]	
25	Framework for the assessment of sustainability				[Eliminated (too broad)]	
26	System dynamics				[Eliminated (too broad)]	
27	Child survival sustainability assessment framework				[Eliminated (not a TMF)]	
28	HIV/AIDS Program Sustainability Analysis Tool				[Eliminated (not a TMF)]	
	Total score across TMFs	19	29	13		

*TMF* theory, model, and/or framework; *T-CaST* TMF Comparison and Selection Tool

Table 3 shows our evaluation of the relevance of the TMFs for conceptualizing sustainment. As can be seen from the bottom row in Table 3, compared to other rating criteria, the TMFs performed best on criterion 2, inclusion of meaningful, face-valid explanations of proposed relationships (total T-CaST score across TMFs, 29). Notably, however, the dynamic sustainability framework only scored 1 on this criterion because the framework shifts between empirical statements about how sustainability occurs and normative ones about what change agents should do to sustain EBPs.

The TMFs performed worst with respect to criterion 3 (including sustainment as an outcome). For example, normalization process theory, which received a score of 1 on this criterion, proposes face-valid relationships among constructs, but the proposed antecedents to sustainment were difficult to distinguish from sustainment itself.

The TMFs also showed inconsistencies in their explanations of how constructs influence sustainment and/or each other (criterion 1; total T-CaST score across TMFs, 19). Several TMFs (e.g., open systems theories, organizational learning theory, complexity theory, and ecological theories) lacked discrete constructs that might be operationalized as antecedents to sustainment.

## Discussion

In our review of 9 reviews, we found that TMFs are underused in sustainment research. This finding parallels previous reports of the underuse of TMFs in implementation science more broadly [38–40]. In most of these reviews, which spanned multiple disciplines and topics, fewer than half of included studies reported using a TMF to conceptualize sustainment. This may reflect studies’ focus on understanding whether an intervention was sustained, rather than determinants of sustainment [26]. As the field shifts from accumulating evidence of poor EBP sustainment to understanding determinants of sustainment, lack of TMF use in sustainment studies represents a missed opportunity to realize the benefits of using TMFs, including their potential to advance a shared understanding of how and why EBPs are sustained [14]. Among those studies that reported using a TMF, there was little convergence on which of the 76 TMFs that we identified in sustainment research to date were used. Indeed, 48 studies which used a TMF did not appear in any of the other studies. This lack of convergence may contribute to a fractured understanding of sustainment across studies, settings, and fields and may retain concerns regarding synonymy and polysemy. Improved reporting and use of TMFs across multiple studies should improve understanding of this critical topic.

Of the 28 TMFs (Table 2; TMFs 1–28) used more than once across the 648 studies, 11 TMFs received a T-CaST score of 4 or 5 out of 6 possible points, suggesting potential relevance to understanding sustainment. Nonetheless, the information underlying these quantitative scores warrants some qualitative exploration. We found that sustainment was seldom the outcome of interest in the TMFs used in sustainment studies. This finding may reflect a shortage of TMFs that specifically target sustainment, lack of researcher’s familiarity with sustainment-focused TMFs, doubts about their utility, or a preference among researchers for broader TMFs. Whatever the cause, the lack of attention to sustainment as the outcome of interest limits the TMFs’ ability to advance our understanding of sustainment. Our understanding of sustainment may be enhanced by converging upon TMFs that include sustainment as the outcome of interest or explicitly acknowledging the limitations for sustainment research of TMFs that include a different outcome. Few of the TMFs explained the mechanisms through which included constructs influence sustainment. Without a strong explanation of the causal pathways that lead to sustainment, the TMFs make limited contributions to identifying and developing potential sustainment strategies. Identifying causal pathways that lead to sustainment implies conceptually sound sustainment strategies. For example, a TMF that suggests that organizations are subject to pressure from norm-setting institutions to sustain a particular practice implies influencing institutional policy as a potentially high-leverage strategy. Further, despite evidence suggesting that some conditions are unique in influencing sustainment, few TMFs distinguished determinants of sustainment from determinants of other, related outcomes (e.g., adoption, implementation) [41].

Although 11 TMFs scored fairly well based on our criteria, each had significant limitations. For instance, normalization process theory (NPT), which describes the social processes leading the routinization of EBPs, explains the relationships among included constructs; however, raters had difficulty disentangling NPT’s concepts of “integration” and “embeddedness” from its core constructs (e.g., coherence, cognitive participation, collective action, reflexive monitoring). Although normalization in NPT is related to (or perhaps even synonymous with) sustainment, articles that presented NPT did not offer a clear conceptual distinction between “integration” “embeddedness” (i.e., sustainment) and implementation. Another class of TMFs, open systems theories, which broadly propose that organizations are strongly influenced by their environments, offered a meaningful, face-valid explanation of sustainment. However, open systems theories did not include discrete constructs, thereby limiting our ability to operationalize or falsify the theory [14]. Further, open systems theories are an umbrella that encompasses several theories, not a singular TMF.

Institutional theory [42–44], which had the highest T-CaST score (5/6), proposes that organizations sustain practices in response to three key pressures related to maintaining legitimacy: mimetic (e.g., mimic other organizations’ behavior), coercive (i.e., meet expectations from organizations providing critical resources, including funds and legal permission to operate), and normative (e.g., act in accordance with professional norms). In other words, the institutional theory suggests that organizations may be more likely to sustain practices if they are under mimetic, coercive, or normative pressure to do so. Further, the institutional theory suggests several moderators of the influence of mimetic, coercive, and normative pressure on organizations’ sustainment of practices. For example, the institutional theory suggests that organizations may be more inclined to mimic other organizations (i.e., sustaining or failing to sustain a practice) in the face of uncertainty and that organizations may be more inclined to cede to coercive pressure from organizations on which they are more dependent.

Understanding of sustainment may be enhanced by understanding the role of mimetic, coercive, and normative pressures and potential moderators that institutional theory proposes. It may also be enhanced through testing strategies for sustainment that the institutional theory suggests: Sustainment may be promoted by assessing the pressures to which an organization may be subject with respect to sustaining a given EBP and then leveraging pressures for sustainment and deflecting those for discontinuation of the EBP.

Despite its potential contribution to understanding sustainment, institutional theory offers limited insight into potentially influential factors at levels other than the outer setting, including the inner setting and individual levels. To some extent, neo-institutional theory [45] may address this limitation. For example, neo-institutional theory suggests that individuals make choices because they see no alternative. Psychological theories, such as theories concerning habits [46], may also offer insight into individual-level determinants of sustainment. Another important critique of the institutional theory is that the institutional pressures that it conceptualizes may be sufficient for superficial organizational change, but sustaining practice may require more intrinsic motivations [47, 48]. The limitations of the institutional theory—or any given TMF—suggests that combining multiple relevant TMFs may be an optimal approach to conceptualizing a construct as complex as sustainment.

Notably, the TMFs that appear to be most relevant to sustainment focus on social dynamics among individuals and organizations. Although sustainment is often inextricably linked with economic resources for delivering EBPs, economic theories for explaining sustainment did not emerge in this review [8]. Indeed, stable financial resources are necessary for sustainment, but our findings highlighting the importance of social processes and relationships suggest that economic and rational choice theories are likely insufficient for explaining whether and how EBPs are sustained in such complex care settings.

A limitation of our study is that there may be other TMFs that are relevant for understanding sustainment but have not yet been cited in healthcare sustainment research. For example, structuration theory [49] explains how social systems create and reproduce structures that uphold and discontinue EBPs. The structuration theory has the benefit of a multilevel orientation to understanding EBP sustainment. Similarly, the contingency theory suggests that EBP sustainment depends on conditions that incentivize sustainment in the face of competing courses of action. Future efforts should assess the relevance of other potentially relevant TMFs for conceptualizing sustainment. And, as the evidence base around sustainment grows, and TMFs are more widely used to support it, the field may benefit from identifying examples of sustainment studies that have effectively applied TMFs.

In this study, we treated TMFs as distinct although we recognize that there are areas of overlap of constructs across TMFs. Identifying overlap of constructs across TMFs may be aided by ongoing efforts to assess the validity and reliability of measures of sustainment-related constructs [50]. Further, as a review of reviews, our study relied upon the information that included the review provided by the authors regarding how TMFs were used in included studies. The degree to which TMFs were used can vary from mere citation to rigorous testing and linkage to specific constructs. Although this level of detail was beyond the scope of our study, we acknowledge the significance of this area for future research. Relatedly, despite the potential for reviews of reviews to leverage existing reviews to develop new knowledge, it is possible that our review of reviews [51, 52] was not entirely comprehensive, and we did not independently review methodological quality.

Another limitation of our study is that, with the exception of the studies cited by Shigayeva and Coker (2015) [17], we did not independently evaluate studies included in the remaining eight reviews. Thus, our estimate of the proportion of studies citing TMFs relies on these reviews’ findings, and the number of studies that we report may include duplicates. Consequently, for the proportion of studies citing TMFs, both the denominator and the numerator may be overestimates; however, the proportion that we report is likely valid. Although we documented assessments of quality conducted by authors of included reviews in Table 1, we did not independently evaluate the quality of published reviews. To the extent that extant reviews’ quality is limited, our findings may not accurately reflect the use of TMFs or their relevance for sustainment research. Nevertheless, our work represents an effort to consolidate existing knowledge to answer novel questions as implementation scientists increasingly appreciate the importance of sustainment.

We used T-CaST to rate the performance of TMFs identified in the review from our own perspectives. Future work should evaluate the relevance of sustainment TMFs from the perspectives of users; users’ perspectives of TMFs’ relevance for their work (e.g., whether they use TMFs for data collection and/or analysis) may enhance our understanding of TMFs’ contributions to conceptualizing sustainment.

## Conclusion

Leveraging published reviews of sustainment studies, we identified institutional theory as a promising TMF for advancing our understanding of sustainment. Incorporating theories that meet the criteria that we advanced above has the potential to promote shared understanding of EBP sustainment. Shared understanding of EBP sustainment will be enhanced by repeatedly applying a few sustainment theories. To limit the synonymy and polysemy that has fractured our understanding to date, we must also agree upon operational definitions of included constructs. Then, we may be able to compare the performance of selected theories and understand the implications of sustainment research for identifying promising sustainment strategies.

## Data Availability

Data abstracted for this review as a supporting file.

## Abbreviations

EBP: Evidence-based practice
T-CaST: Theory, Model, and Framework Comparison and Selection Tool
TMF: Theory, model, and/or framework
